# Arabic Translation and Linguistic Validation of the SCAR-Q Scale Module

**DOI:** 10.7759/cureus.20468

**Published:** 2021-12-16

**Authors:** Omar Braizat, Nasrin Jafarian, Sequina Al-Saigel, Salma Jarrar

**Affiliations:** 1 Plastic and Reconstructive Surgery, Hamad Medical Corporation, Doha, QAT

**Keywords:** translation research, questionnaire development and validation, plastic and reconstructive surgery, scar appearance, heath related quality of life

## Abstract

Scars are a fairly common complaint in the clinical setting; they can arise from trauma, burns, or after surgical procedures. They can have a detrimental impact on the quality of life of patients. A well-established method of quantifying such an impact is through patient-reported outcome measures (PROM). SCAR-Q is a relatively new and robust questionnaire that was developed and validated by McMaster University. Our study aims to translate SCAR-Q to Arabic. SCAR-Q has a holistic approach to scars and does not target scars of a specific etiology. It has three main components: scar appearance, psychological impact, and symptoms associated with the scar. This translation will enable data collection, analysis, and interpretation from a previously untouched demographic. This article explains the steps taken to develop an accurate and validated Arabic SCAR-Q questionnaire based on World Health Organization (WHO) and The Professional Society for Health Economics and Outcomes Research (ISPOR) guidelines. We were able to methodically produce a validated translation of the SCAR-Q into Arabic that should potentially allow data collection and feedback from a very large segment of the world population regarding the impact of scars on their quality of life.

## Introduction

Scars form when the outer layer of the skin is damaged, and they can take different shapes, forms, and colors. Scars change the natural body appearance and subsequently negatively impact the quality of life (QoL). Patient-reported outcome measure (PROM) and clinical outcome assessment (COA) are essential to comprehensively evaluate, quantify, and compare the impact scars have on the patient’s QoL [1]. SCAR-Q is a PROM that was developed by McMaster University and validated on 731 patients [2]. It attempts to accurately measure the impact of scars on the QoL of patients and is not limited to scars of specific etiologies or anatomic locations; it can also be applied to the pediatric population [3].

According to Ziolkowski et al. [3], multiple tools have been developed in recent years to evaluate the impact scars have on QoL, including multiple clinical outcome assessment (COA) tools. Such tools represent the health care provider’s perspective and not how the patients perceive their scars. SCAR-Q is a patient-reported outcome (PRO) instrument developed based on measuring the patients’ concepts of interest (COI) [4] with three main subscales in mind: appearance, symptoms related to the scar, and the psychological impact of the scar [2]. Scar appearance section probes into how visible and noticeable the scar is. The symptom part investigates how the scar feels, and finally, the psychosocial section inquires about the psychological impact of the scar on the patient [5].

Other PRO instruments exist and include the Stony Brook Scar Evaluation Scale (SBSES), Manchester Scar Scale (MSS), and Visual Analog Scale (VAS), which also assess the nature of scarring to specific body parts. Although the tools mentioned above are popular and extensively used, they tend to be specific to certain etiologies and do not accurately measure the psychosocial impact of scars [6].

We aimed to produce a validated translation of the SCAR-Q into Arabic. Arabic is the fifth most spoken language globally, spoken by an estimated 466 million people. Therefore, an Arabic translation of SCAR-Q will allow data collection from a large demographic, i.e., the Qatari population, the Gulf countries, and possibly all 25 Arabic-speaking states. The translation was carried out in a culturally sensitive manner to suit the needs of the targeted population.

## Technical report

Original SCAR-Q module

After obtaining approval from the developers of the original SCAR-Q [2], we commenced the translation and validation process. As Lorenzen et al. [7] stipulated, we abided by the World Health Organization (WHO) and The Professional Society for Health Economics and Outcomes Research (ISPOR) recommendations during the translation process. This resulted in an accurate and valid translation of the SCAR-Q. Table 1 shows a step-by-step comparison between our translation with the WHO and ISPOR directions [8].

**Table 1:** Table comparing our translation process to WHO and ISPOR guidelines. WHO: World Health Organization, ISPOR: The Professional Society for Health Economics and Outcomes Research

Steps	ISPOR Guidelines	WHO Guidelines	Our translation and validation methodology of the SCAR-Q
1	Preparation – Seeking permission from the people in charge.	Professional translation based on conceptual meaning by independent translators.	SCAR-Q team at McMaster University were contacted, and an official translation license was signed.
2	Forward translation – Translation to the target language by two or more independent translators.		Two independent translators did two forward translations into Arabic.
3	Reconciliation – The forward translations are reconciled into a single translation.		The two translators reconciled the two translations into one translation.
4	Back Translation – The reconciled translation is translated to the target language version.	Back translation is based on cultural and conceptual meaning.	Back translation was completed by a native English-speaking translator fluent in Arabic.
5	Back Translation review – The back translation is reviewed compared to the original language.	Expert panel - bilingual expert panel	Back translation was compared to the original version by the SCAR-Q team, and feedback remarks were provided.
6	Harmonization – The two versions are compared.		
7	Cognitive debriefing	Pre-testing and cognitive interviewing.	Cognitive interviews were performed, and any vague or misunderstood statements were amended.
8	Review of cognitive debriefing results and finalization	Final version and documentation.	The updated version was reviewed and finalized.
9	Proofreading		Proofreading by medical professionals.
10	Final report		The final Arabic SCAR-Q version was approved and generated.

Translational procedure

**Figure 1:**
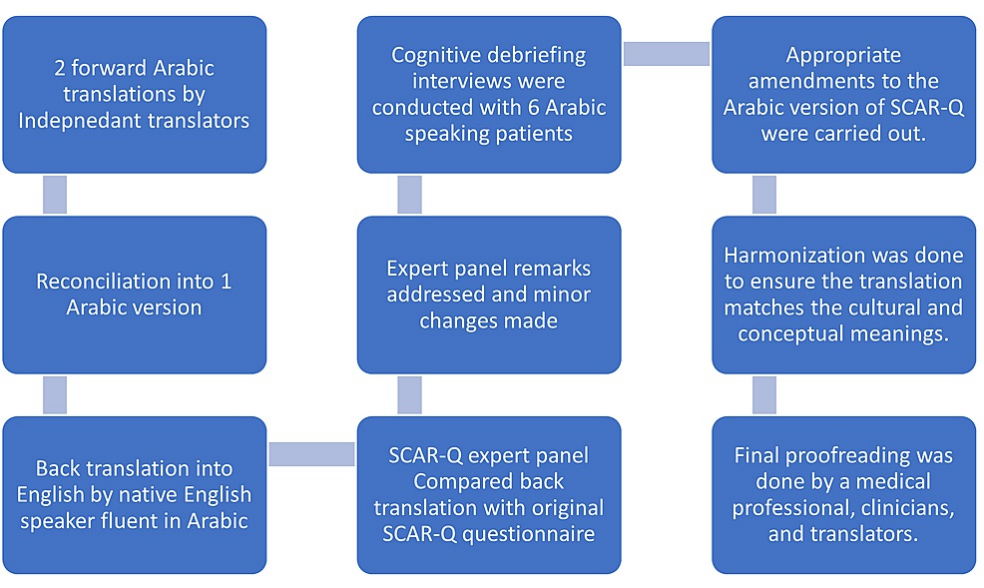
Flowchart outlining the translation and validation steps we followed.

Figure 1 presents a flowchart outlining the steps we followed to create and validate an Arabic translation of SCAR-Q. The team leader obtained a signed translation license with the owners of the SCAR-Q module that modulated the translation process. A team meeting was held, and the SCAR-Q concepts were discussed with the two forward translators. Two native Arabic speakers who are fluent in English performed independent forward translations (English to Arabic). After discussion with both translators, the two Arabic forward translations were reconciled into one version.

Back translation of the reconciled Arabic translation to English was done by a third independent senior plastic surgery resident whose mother tongue is English; she has lived all her life in Qatar and is fluent in Arabic. This physician was blinded to the original English version to avoid any bias during the back translation. The Q-portfolio team held an expert panel meeting. They compared the English version to the back translation and provided feedback remarks and clarification requests. Minor changes were made, and all remarks of the SCAR-Q team were addressed.

Cognitive debriefing interviews were conducted with six patients between the ages of 20 and 40. The male to female ratio was 1:2. All patients were Arabic-speaking Qatari nationals. Recruitment was done through our plastic surgery service. Patients with scars of different etiologies were randomly selected and invited to join the study. Consenting patients were asked to describe any difficulty understanding any word or statement in the final Arabic version of the SCAR-Q module. Their feedback was documented and gathered in an Excel spreadsheet (Microsoft® Corp., Redmond, WA). Two patients found one statement to be unclear: “How does your scar look from different angles?” The Arabic translation did not illustrate the figurative meaning of “angles,” so it was changed to “How does your scar look from different views?”

Harmonization was done by all parties involved in the study; the process evaluated the new translation to ensure it matched the cultural and conceptual meanings. Final proofreading was done by a medical professional, clinicians, and translators.

## Discussion

Scar quality assessment is an integral part of the outcome of all surgical procedures, especially in the field of plastic and reconstructive surgery [9]. Our translation methodology has been previously implemented by many authors [7,10]. After following all the steps recommended by WHO and ISPOR, we developed the Arabic version of the SCAR-Q that is linguistically validated and culturally fitting, similar to the production of the Danish translation of this PROM [7].

Undertaking the process of translating a patient-reported outcome measure warrants a delicate balance between implementing the necessary changes to ensure the questionnaire is well adapted and culturally sensitive and safeguarding the intellectual content of the original version. For this reason, we were adamant about abiding by the WHO and ISPOR guidelines [8]. As demonstrated in Table 1, our translation methodology is more in line with the ISPOR guidelines, which describe a more detailed and meticulous translation process.

ISPOR recommends performing five to eight debriefing interviews [7]; we performed six. Interviewed patients had scars of different etiologies; surgical, traumatic, and burn scars were all included. During the interviews, only two out of the six patients had comments on one statement, which indicates a clear and culturally sensitive translation. Possible limitations of our translation include a relatively small number of debriefing interviews, which does not allow the use of statistical tests that can assess the internal validity of the questionnaire in Arabic, as performed by previous authors [10]. Furthermore, the forward and backward translations were not performed by a professional translator. We, therefore, encourage fellow researchers to utilize the translated Arabic version of the SCAR-Q questionnaire on larger samples for purposes of further validation and data collection on scar related quality of life after different surgical procedures

## Conclusions

The SCAR-Q is an accurate and validated tool that evaluates the effect of scars on the patient’s quality of life. This can aid both physicians and patients in predicting risk factors for decreased quality of life after certain scars. It can also help in surgical planning and scar placement. Moreover, a patient-specific scar treatment plan can be formulated to achieve maximum patient satisfaction. SCAR-Q has been translated into many languages. The most significant value of this validated translation is that it will allow data collection and feedback from a substantial segment of the world population: the Arabic-speaking nations.
